# Network analysis of gratitude messages in the learning community

**DOI:** 10.1186/s41239-022-00352-8

**Published:** 2022-09-07

**Authors:** Masami Yoshida

**Affiliations:** The Faculty of Education, Chiba University, 1-33 Yayoi, Inage, Chiba, 263-8522 Japan

**Keywords:** Collaborative learning, Distributed learners, Exponential random graph models, Gratitude, Online discussion forum, Social network analysis

## Abstract

In pedagogical practice, gratitude is recognised not as an emotion, but as an approach to learning. This study introduced gratitude messages into the academic online communication of university students and specifically examined the community in which students shared their messages with gratitude. This study examined the tendency of message connections and how gratitude messages prompted replies. To elucidate their connections, exponential random graph models (ERGMs) were used. A post-event questionnaire to evaluate gratitude experiences was also administered. Results revealed that 77.3% of the 172 connected messages from 123 students involved gratitude. When the post-event questionnaire results were examined using an ERGM, the score effects on increasing message connections were found not to be significant. The most prominent indication was a higher level of significant propensities to make mutual connections. The homophily of the message content was found to have a significant propensity to increase connections. The ERGM results and a review of messages revealed that students expressed gratitude for being both benefactors and beneficiaries of gratitude messages, which confirmed their prosocial behaviour.

## Introduction

When people receive benefits from others, they often experience feelings of appreciation and gratitude (Algoe et al., 2016). Newer pedagogy posits gratitude not as an emotion, but as an approach to learning and teaching that actively involves the acknowledgement of what has been received and the conscious action of wanting to give back in some manner (Howells, 2014). Gratitude is not a natural trait. In fact, it is a learned and sometimes effortful process that requires a certain degree of introspection and reflection (Wilson & Harris, 2015). People often underestimate the benefits and overestimate the costs of expressing gratitude, thereby creating a misplaced barrier hindering expressions of gratitude (Kumar, 2022). Therefore, the gratitude practice should be chosen at times when students are intentionally engaging in conversations with others about positive events, experiences, or outcomes in which their responses are coded on the good dimension (Fredrickson, 2004). Gratitude practices applied in school settings improved positive social behaviour (Bono et al., 2019). Such positive feelings would encourage them to view others more positively and therefore to show more prosocial actions (Grant & Dutton, 2012). Gratitude studies in education have revealed that expressions of appreciation enhance students’ motivation to engage in prosocial behaviour and subsequently engender stronger relationships and increased engagement within school communities (Freitas et al., 2011). Emphasis on gratitude is crucially important for understanding the mechanisms underlying curriculum success (Layous & Lyubomirsky, 2014).

Earlier reports have described that the cultivation of gratitude serves as a route to facilitate effective learning (Valdez et al., 2022). Gratitude is regarded as one of the seven strengths of character (i.e., gratitude, grit, zest, self-control, optimism, social intelligence, and curiosity) that promote students’ success in academic environments (Tough, 2011; Wilson & Harris, 2015). In the lesson, what is known as ‘a state of preparedness’ approach is sometimes used: students are asked to prepare themselves before academic learning to become aware of their gratitude (Howells, 2004). In this lesson, the positive effects of a grateful attitude were directed by a teacher. According to Howell (2012), students who are ungrateful about a lesson exhibit limited ability to think, concentrate, incorporate information, or see the value of learning. In contrast, when students receive a lesson with gratitude, they are more engaged, focused, and motivated to exert efforts at learning. Gratitude has been used as a learning tool for university students, with evidence that students experienced increased engagement, greater connection to the topic, deeper understanding of content, and increased motivation when they expressed gratitude more frequently.

Gratitude is positively associated with the academic and autonomous motivation of students (King & Datu, 2018; Valdez & Chu, 2020). Examinations of college students have indicated that practising gratitude within the learning environment is associated with increased concentration ability and resilience when facing difficulties in learning (Wilson, 2016). Another positive effect of gratitude has been observed in classrooms. Gratitude has led to a more positive and calmer classroom atmosphere, better-behaved students, and a greater willingness of students to concentrate their efforts on learning (Froh et al., 2008; Wilson & Harris, 2015). Consequently, gratitude inspires engagement in the learning process. For example, whereas students realize the need to follow the lesson subjects and expectations of students, grateful students expand those expectations by pausing to wonder, asking big questions, thinking ethically, and exercising social graces (Wilson & Foster, 2018).

Online communication has been found to be as authentic as offline interaction (Locher, 2010). Even though online interaction is quite natural and spontaneous, the technology allows writers to plan, organise, and check their messages before they are sent, giving them the opportunity to be pragmatic, clear, and polite (Flores-Salgado & Castineira-Benitez, 2018). Gratitude has a slight but significant positive effect on negative emotions when online, wherein individuals with higher gratitude can change their level of negative emotions quickly (Greetham et al., 2011). In online peer-review activities of students, gratitude was proven to be extremely useful in feedback, which included praise, error acknowledgment, or intention of revision (Misiejuk et al., 2021). Because activation and gratitude in a peer can improve group relatedness and can stimulate mastery in in-person groups rather than performance goals, the online group has the opportunity to show more persistence than expected in individual situations (Avry et al., 2020). Although online communication has a weaker association with social interaction, it has a stronger positive association with dyadic contact, rationality, and reciprocal feedback than impersonal communication (Murphy & Sashi, 2018). Online gratitude messages can catalyse academic motivation and cognitive engagement through interpersonal mechanisms (Valdez et al., 2022). Even online users supported by a virtual peer were more inclined to offer social support to their benefactor (Collange & Guegan, 2020).

Effects of gratitude messages as an educational tool extending to the personal, group, and online community practice in educational field have been demonstrated. Despite the growing evidence indicating the potential academic gains associated with cultivating gratitude in schools, specific grey areas have remained unaddressed in earlier research (Valdez et al., 2022). Particularly, no analysis has assessed online learning environments in which gratitude messages were disseminated.

## Methodological framework

### Measuring gratitude

Earlier psychological studies have used questionnaires and surveys to quantify gratitude (Allen, 2018). A Questionnaire of Gratitude Adjective Checklists was developed to ascertain whether students experience gratitude as an emotion, mood, or disposition, depending on the timeframe specified in the relevant set of instructions (McCullough et al., 2002). The Gratitude Questionnaire 6 (GQ-6), a six-item self-assessment scale, was developed to measure an individual’s self-reported level of gratitude as an affective trait or disposition (McCullough et al., 2002). Actually, GQ-6 includes assessments of four qualities of gratitude: intensity, frequency, density, and span. It rates the degree of gratitude among students, from highly grateful students to less grateful students (Froh et al., 2011). The Gratitude, Resentment and Appreciation Test measures dispositional gratitude by analysing the relation between the trait of gratitude, feelings of gratefulness, and happiness (Watkins et al., 2003). A recently proposed questionnaire method, the Expression of Gratitude in Relationships Measure (EGRM), includes three items that measure gratitude in relationships (Lambert et al., 2010). The items are the following: a) I express my appreciation for the things that my partner does for me; b) I let my partner know that I value him/her; and c) When my partner does something nice for me, I acknowledge it. Using this questionnaire, students rate how often they engage in these behaviours on a five-point scale (1 = *never* to 5 = *very frequently*). The numbers are combined into a single expression of gratitude (Allen, 2018). This method is better suited to measuring gratitude in a learning community where a student has an increased perception of communal strength.

These questionnaire methods have been used in earlier studies to measure gratitude experiences among students, but they have some biases because the students were asked about their opinions of gratitude after the communication experiment was completed. Additionally, most traditional self-reported questionnaires only assess relationships among a benefactor, a beneficiary, and at most, a third party (Chang et al., 2012).

### Social network analysis

An earlier report described that on-campus students expressed resistance to incorporating peer relationships into their learning activity, but online students reported effects on communication with getting to know students (Butz & Stupnisky, 2017). Consequently, the use of innovation has changed the way people communicate and exchange information (Schwade & Schubert, 2018). For this investigation, the innovation addressed is all about connectedness, ideation and collaboration between individuals, sharing reflections and findings, and realising potential together. Students' feelings of relatedness (i.e., feeling connected to others) are crucially important for success in any learning environment (Butz & Stupnisky, 2017). Superior capacity is formed when distributed individuals work together with the technology assistance to gather new insights, ideas and information to solve an issue (Gul et al., 2021).

The current study introduces and then examines a holistic view of the online community and examines gratitude in relation to the attributes of each member student and other communication connections from the perspective of the connection structure. Social network analysis (SNA) has made it possible to analyse and visualise connections of an online community by monitoring the online environment (Riquelme et al., 2019). An SNA facilitates investigation and interpretation of communication phenomena and represents how one individual interacts with another. Networks are representations of relational data. After the links are first converted into nodes (students) and edges (online messages), the theory of social networks examines the social and structural processes related to the formation of connections in social networks (Mamas et al., 2020). According to the SNA literature, influence and prestige within a social network are frequently associated with an individual’s degree of connectivity within that network, and with the individual’s membership in many subgroups (Vercellone-Smith et al., 2012). Network connections tend to form based on perceptions of social similarity (homophily) and the common attributes of the members (Spillane et al., 2012).

### Exponential random graph models

This study adopted an innovative method for SNA using exponential random graph models (ERGMs), which are statistical models used in an SNA to observe the underlying mechanisms of structure production (Amati et al., 2018). An ERGM can provide and test inferential hypotheses based on their exponential distribution. The individual covariates in the network (i.e., user attributes) and network structural properties (i.e., reciprocity or transitive triplets) are useful to predict properties of the entire network (Van der Pol, 2019). These elements are involved in an analysis as independent variables. An ERGM summarizes the measure of network statistics of social graphs using the formula presented below.

$$P_{\theta } (G) = ce^{{\theta_{1} z_{1} (G) + \theta_{2} z_{2} (G) + \cdots + \theta_{p} z_{p} (G)}}$$

The probability *P* of the network *G* provides a value of 0–1; it is the sum of the network statistics (*z* in this expression), weighted, as with regression, by model parameters (*θ*) inside an exponential, where *c* is a normalising constant (Lusher et al., 2013). Each *θ*_*i*_* Z*_*i*_ (*G*) is called a ‘term’ to represent the network statistics. One term adds one network statistic to the model.

**Fig. 1:**
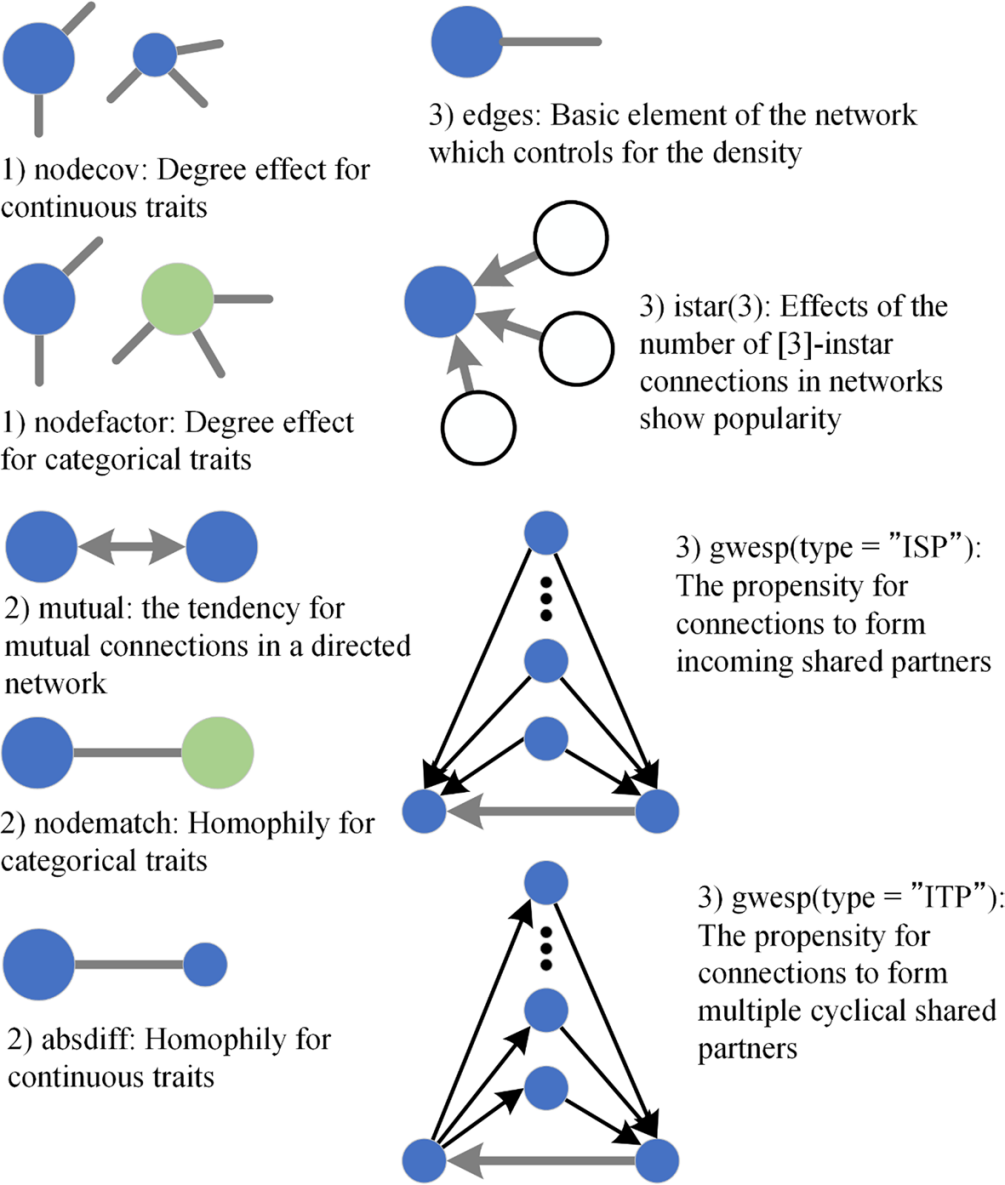
Diagrammatic guide to key terms used for this study. The number at the top of each term denotes the related covariate: 1, node-based; 2, dyadic; 3, structural

Figure 1 presents examples of some ERGM terms. From a practical perspective, the terms fit the three broad categories of node-based, dyadic, and structural covariates (Silk & Fisher, 2017).

#### Node-based covariates

Node-based covariates explain differences in edge values as outcomes of the attributes of the nodes themselves. For example, the term ‘nodecov’ denotes whether students with higher scores for a continuous variable displayed a tendency to nominate more connections. The term ‘nodecov.access’ calculates propensity based on students’ access times to the discussion forum.

#### Dyadic covariates

The dyadic covariate model examines how relationships between individuals affect edge values. The term ‘absdiff’ denotes homophily in the network based on a continuous variable. The term ‘mutual’ represents the propensity based on how many connections are reciprocated. The term ‘nodematch’ stands for whether students have a propensity to nominate connections with those with whom they share a particular attribute. In other words, it denotes homophily in the network based on a categorical variable.

#### Structural covariates

Structural covariates are those aspects of the network topology that are expected to affect edge formation and which are inferred as encompassing multiple levels of complexity. The most basic structural term is a measure of edge density. Increasingly complex structural terms can be incorporated. These define the dependency structure used within the model to elucidate how the presence or absence of edges affects nearby edges. For example, the term ‘istar(3)’ in Fig. 1 can measure the likelihood of popularity, which comprises changes in three incoming connections.

Calculations of the ERGM are conducted using Markov Chain –Monte Carlo methods (Handcock, 2003; Snijders, 2002). Estimates of the statistical parameters are based on an underlying simulation for which many networks are created to reflect the particular model being tested (Luke, 2015). Estimation of the maximum likelihood of each parameter is calculated by generating values for all parameters that centre the distribution of each parameter fitted to the observed network data. The parameter estimation is conditionally dependent on other covariates included in the model (Lusher et al., 2013).

Although research into community networks among university students has been reported in the literature (Fujiyama, 2020; Kornienko et al., 2014; Yon et al., 2021), no report of the relevant literature has described a case study that investigates the effects of students’ gratitude messages using ERGM. This study is the first to identify determinants associated with structural characteristics of a learning community using this method.

### Education for sustainable development as lesson content

The content of the target course for this study was Education for Sustainable Development (ESD). The United Nations 2030 Agenda comprises 17 sustainable development goals (SDGs) to be met by 2030 as a matter of priority (United Nations General Assembly, 2015). These 17 SDGs, which are listed in the preamble of the declaration, promote sustainable development in an integrated manner (United Nations Educational, Scientific & Cultural Organization, 2020; UNESCO). UNESCO and other international organisations recommend empowering youth to implement the SDGs (Albareda-Tiana et al., 2018). Universities have undertaken efforts at implementing ESD curricula into their frameworks to achieve the SDGs and to develop student competencies (Tejedor et al., 2019).

## Research Questions

This study investigated the online activities of university students who shared messages on an online discussion forum. The following four research questions were posed:

- *RQ1:* What attributes of interlocutors account for students making connections?
- *RQ2:* What are online community characteristics? What structural connections of the network tend to emerge?
- *RQ3:* How do messages of gratitude appear? To what extent are these messages deployed in the network?
- *RQ4:* How do messages with gratitude prompt reply messages?

## Methods

### Target course

The Japanese university that organised this study, by opting for a strategic approach to internationalisation, offered an ESD course for students. This course, taught in Japanese, was an introductory course for students. It was offered online because the spread of COVID-19 made on-campus classes prohibitive. Therefore, Moodle was used as the platform for online lessons.

### Participants and ethics

The target students were first-year undergraduate students. The course was offered during their first semester at the university. Therefore, the students had little opportunity to develop friendships before engaging in the online discussion. Drawing from two faculties (engineering and nursing), 123 first-year undergraduate students were recruited for this study. Student communication in a Moodle discussion forum was monitored for a week in May 2021. All procedures performed for this work were done in accordance with the ethical standards of the institutional and national research committees. All complied with the 1964 Declaration of Helsinki and its later amendments. Informed consent was obtained from all students involved in data collection processes.

### Lesson process

The target course was designed to promote the exchange of views to understand the disparity among countries that intend to practise the ‘systems thinking competency’ of the ESD key competencies (UNESCO Division for Inclusion, Peace, & Sustainable Development, Education Sector, 2017). Global learning requires adoption of relational perspectives. Earlier reports have described that students learn more efficiently when lessons involve collaboration (Luckie et al., 2012).

**Fig. 2:**
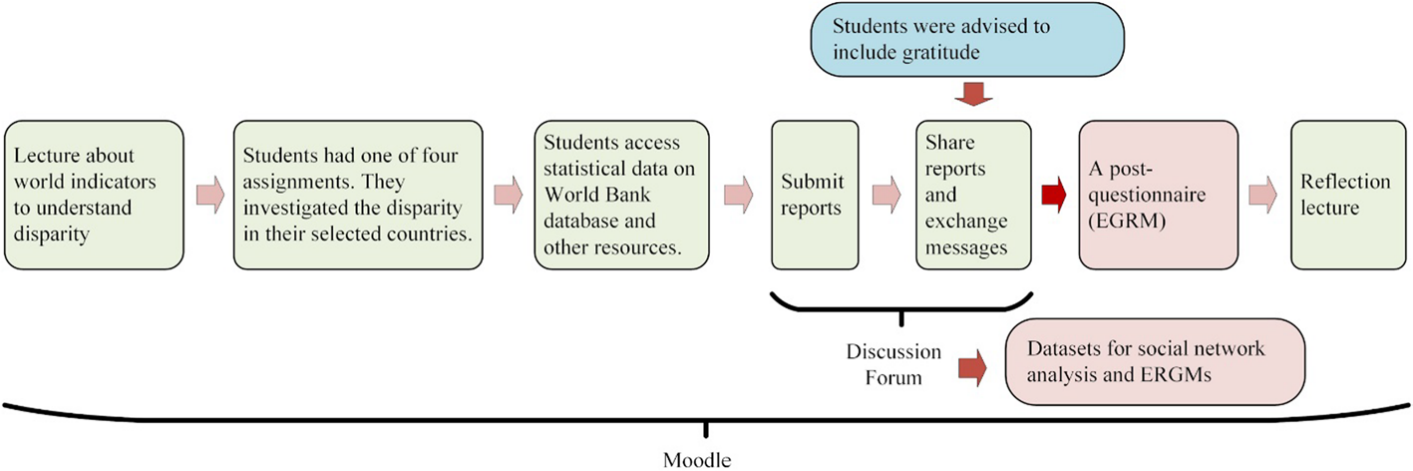
Overview of lesson process

The lesson was conducted according to the following procedure (Fig. 2).

After an introductory lecture, each student was assigned one of four questions (Table 1). Each question comprised two indicators for which students were able to obtain detailed longitudinal data through the World Bank’s online database. They had to select the country and finalise the comparative year periods for the two indicators to collect data. The students were instructed to consider potential confounding factors when explaining the relations among the indicators. They also had to collect additional information related to each selected country to explain trends in the indicators.

**Table 1:** Questions used for the assignment (translated by the authors)

Number	Assignment
1	Select a country with a GDP per capita (PPP) of $10,000 and more. Compare the Gini coefficient and employment in industrial sectors as a proportion of total employment over time. Investigate and explain the disparity in the country
2	Select a country with a GDP per capita (PPP) of $10,000 and more. Compare the Gini coefficient and the ratio of government debt repayments in foreign currency income over time. Investigate and explain the disparity in the country
3	Select a country with a GDP per capita (PPP) of $10,000 and more. Compare the Gini coefficient and the enrolment rate in higher education over time. Investigate and explain the disparity in the country
4	Select a country with a GDP per capita (PPP) of $10,000 and more. Compare the Gini coefficient and individual internet usage rate over time. Investigate and explain the disparity in the country

*GDP* gross domestic product, *PPP* purchasing power parity

After completing self-regulated learning via the internet and university library, they were asked to submit a report through a Moodle discussion forum. The reports submitted by the students were then shared. The students were allowed to post comments and messages, and to exchange ideas with others continuously.

### Introduction of messages of gratitude

Because all information for effective learning should be bundled (Stasser & Titus, 2003), an online discussion forum was introduced for this study to enable the exchange of information within the learning environment. As a way of encouraging students to post comments and messages, they were advised to offer words of gratitude to their peers. After communicating in the discussion forum, the teacher offered a lecture to provide a deeper understanding of disparity. A post-questionnaire using EGRM was conducted.

### Data collection

The messages posted by the students were collected. The following data were included in the dataset: scores (1–10) for quantifying disparity as assessed by a course instructor, number of characters in the messages, number of discussion forum access iterations, number of messages that included gratitude, number of links or references in messages, number of figures in messages, gender (male or female), name of the target country, question number (1–4) for the assignment, and number of messages posted.

For data analysis, NodeXL Pro, a social network analysis toolkit, was used to organise the data, calculate metrics, and generate social graphs. Continuous analyses were processed by ERGM using the ‘statnet’ and ‘stargazer’ packages for R (ver. 4.0.3; R Foundation).

## Results

### Result of EGRM

Results of the EGRM questionnaire indicated that the students had a positive experience of exchanging gratitude messages in the study (*M* = 12.75, *SD* = 2.15). In earlier studies using EGRM, a case with similar result was reported (*M* = 12.66, *SD* = 0.30) in which spousal gratitude was valued (Barton et al., 2015). Results of the current study then proved that the students experienced greater feelings of thankfulness and appreciation in the scholarly discussion environment.

### Emerged network

**Fig. 3:**
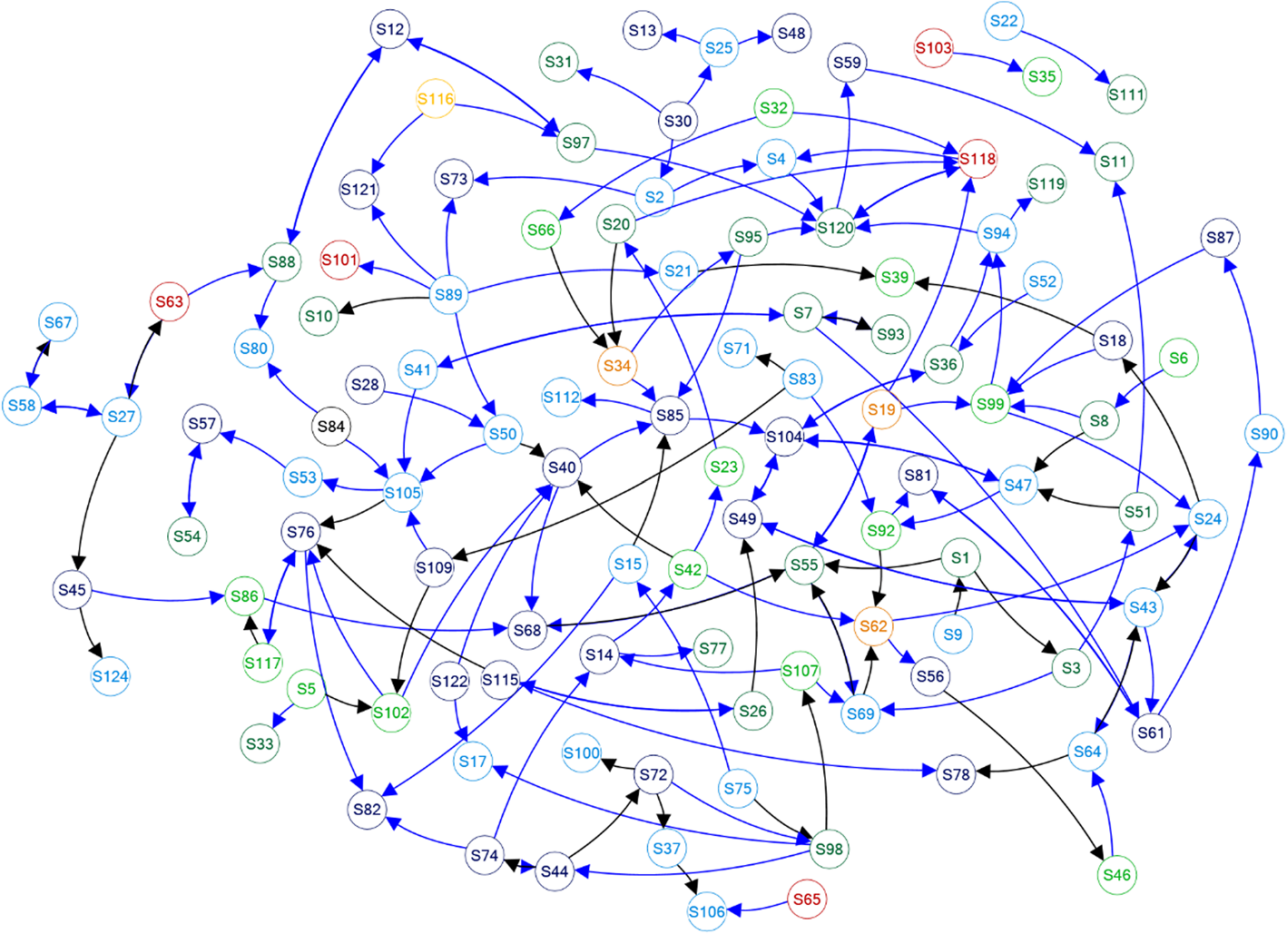
Social graph of the academic community. Blue edges represent messages of gratitude, with the node colour representing countries about which students have submitted reports. The node labels are identifiers of students

Figure 3 portrays a social graph of the network that emerged to represent the community. In the network community, 300 edges (messages), 170 unique edges (non-duplicated messages), and two duplicated edges were detected. 111 nodes had connections to another student. The maximum number of nodes in a connected component was 107.

**Fig. 4:**
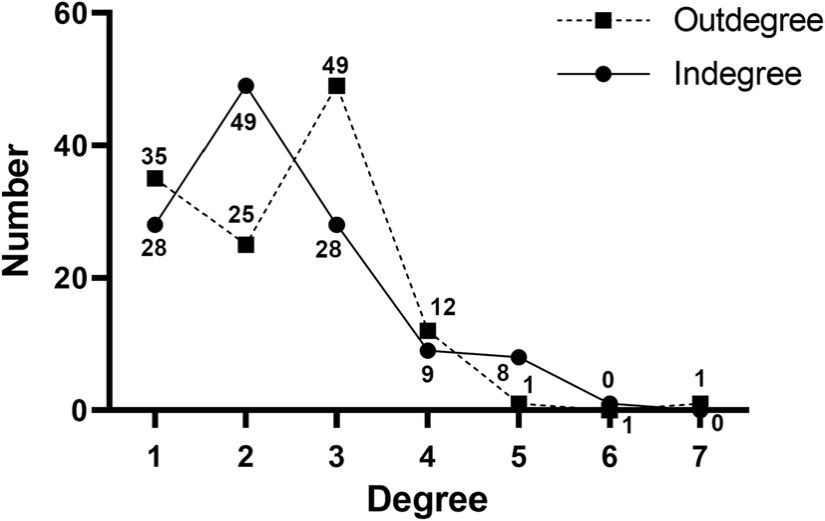
Degree distribution of the community

Figure 4 shows the distribution of indegree and outdegree of the network. The average outdegree (the mean of outward connected messages posted by student) was 2.37. This number was regarded as having active participation in the community. An earlier reported case study examining student communication in an online discussion indicated that students were reluctant to participate (Watson et al., 2017, p. 282). In this case, they wanted to take the course to increase their knowledge of the subject matter, but did not want to become involved in arguments. However, the students in our study were actively posting messages. The number of connections that developed surpassed the earlier result.

Figure 4 also shows that they selected widely diverse students for communication. The median indegree (*Mdn* = 2) is smaller than the median outdegree (*Mdn* = 3). No noticeable concentration of connections is depicted in Fig. 3, suggesting the need to investigate factors that drive students to make connections.

### Results of the ERGM by category

Table 2 presents results of the ERGM by category.

**Table 2:** Results of maximum likelihood estimation on ERGM parameters

Term	Dependent variable: Estimate		
Parameter	Node-based	Dyadic	Structural
edges Messages	− 6.475*** (0.889)	− 5.097*** (0.222)	− 4.229*** (0.110)
nodecov.score Evaluation score by an instructor	0.056 (0.046)		
nodecov.character Number of characters in messages	− 0.0001 (0.0001)		
nodecov.access Number of discussion forum access iterations	0.005* (0.003)		
nodecov.gratitude Number of messages with gratitude	0.218*** (0.053)		
nodecov.EGRM Score of questionnaire	0.020 (0.030)		
nodecov.link Number of web links in messages	− 0.003 (0.031)		
nodecov.fig Number of figures in messages	0.070 (0.063)		
nodefactor.gender.female Female students	− 0.109 (0.151)		
mutual Reciprocal messages		3.184*** (0.298)	
nodematch.country Same country		0.994*** (0.267)	
nodematch.question Same question number		0.926*** (0.142)	
nodematch.gender Same gender		0.376** (0.170)	
absdiff.score Homophily in evaluation score		− 0.114** (0.060)	
absdiff.character Homophily in character count		− 0.0001 (0.0001)	
absdiff.link Homophily in number of web links		0.044 (0.031)	
absdiff.fig Homophily in number of figures		− 0.088 (0.075)	
absdiff.access Homophily in number of discussion forum access iterations		0.003 (0.003)	
absdiff.gratitude Homophily in number of gratitude messages		0.129** (0.065)	
absdiff.EGRM Homophily in score of questionnaire		− 0.059 (0.038)	
istar(3) Incoming 3 edges			− 0.046 (0.072)
gwesp.ISP Incoming shared partner			− 0.535 (0.677)
gwesp.ITP Multiple cyclic closure			0.335* (0.201)

Upper, parameter estimate value; lower, parameter standard error

*p < 0.1; **p < 0.05; ***p < 0.01

#### Node-based covariate analysis

The results of this analysis are incorporated into the interpretation based on the node attributes of the students. Significance was found in the propensity of ‘nodecov.access’ and ‘nodecov.gratitude’. No significance was found in the propensity for connections in other terms: ‘nodecov.score’, ‘nodecov.character’, ‘nodecov.EGRM’, ‘nodecov.link’, and ‘nodecov.fig’. Therefore, we found that the scores of the questionnaire were not associated with the propensity for connections. Although a report of an earlier study described that female respondents showed higher levels of gratitude (Fredrickson, 2004), their propensity to form more connections was not observed in this study, based on the ‘nodefactor.gender.female’ result.

Whereas the baseline of a connection probability was calculated as 0.00154, the log odds of the connection increased when ‘nodecov.gratitude’ was included: The probability is 0.00191. The influence was found to be significant, but the effect on the community by those with multiple messages of gratitude was not strong.

#### Dyadic covariate analysis

In the network of this study, 22 of the 170 unique edges were reciprocated. The number of mutual connections was compared with generated random networks of the same density in the calculation of the ERGM. The effect was strongly positive and significant (*Estimate* = 3.184, *P* < 0.01), indicating more mutual connections in this network than one would expect from a random network with 170 unique edges.

The terms ‘nodematch.country’ and ‘nodematch.question’ had higher coefficients with significant propensities for nominating interlocutors when they posted messages about the same country or the same question number. Being the same-gender related to the term ‘nodematch.gender’ also had a significant and positive effect on propensity to nominate a peer, although the contribution to the estimated probability values was small. Connections from students with similar assignment answers scores had fewer incoming connections than pairs with different scores. This finding was shown by the significant and negative coefficient of the term ‘absdiff.score’, but the coefficients were small. The effects were likely to be slight.

No significance was found in the propensities of the other terms: ‘absdiff.character’, ‘absdiff.link’,’absdiff.fig’, ‘absdiff.access’, or ‘absdiff.EGRM’. Therefore, no homophily was found in scores of the questionnaire. The homophily in gratitude message numbers ‘absdiff.gratitude’ had an increasing propensity; connections increased more in active communicators than in students with great gratitude experience perception. Although the baseline of a connection probability was calculated as 0.00608, the log odds of the connection are greater when ‘mutual’, ‘nodematch.country’, ‘nodematch.question’, and ‘absdiff.gratitude’ are included. The probability was 0.534.

#### Structural covariate analysis

For the relational structure, the term ‘gwesp.ITP’ calculated the propensity for connections to form multiple cyclical shared partners (see Fig. 1) where a student has multiple incoming two-path connections. Although a significant propensity and propinquity of two nodes was observed in the ‘gwesp.ITP’, no status difference was found between a student and interlocutor. The result also supports strong homophily in the attributes of messages that appeared in the dyadic covariate analysis. Conversely, whereas the term ‘gwesp.ISP’ of the propensity for the incoming shared partners showed no significance, multiple popularity was not observed for any student pair on the network. In addition, 51 three-istar connections were counted on the network (‘the term ‘istar(3)’ is a cue to the popularity of a student) but were not significant. Whereas Section ‘2. dyadic covariate analysis’ shows that students preferred to make reciprocal connections based on the message content, these results implied that student behaviour in monitoring the communication of others was based on the contents. These students monitored students who sent them messages and identified the third student who sent messages to their messenger. The meaning of significance in ‘gwesp.ITP’ is that they sent communication messages to this third student. Because the baseline of a connection probability is detected as 0.0144, the log odds of the tie increased when ‘gwesp.ITP’ was included and the probability was 0.0200. Therefore, the structural covariates have little effect.

In the current study, seven terms in the dyadic covariates analysis showed significant propensities to increase connections, as shown in Table 2. The interrelationship of students in the network was meaningful and communicative. Particularly, the terms that fit into the dyadic covariates showed that the students in the network are prone to make connections with the same attributes, whereas the effects on node-based covariates and structural covariates appeared less frequently. Earlier research results have indicated that exchanging gratitude with both partners benefits close relationships, but one partner with high gratitude and another with low gratitude disrupts the mutual well-being of both partners (McNulty & Dugas, 2019). This concept that the partner’s perceived responsiveness was a critical component of close collaborative relationships explained the gratitude in the mutual messages shown in our results.

## Discussion

### RQ1: What attributes of interlocutors account for students making connections?

The result of ERGM calculation identified the role of attributes in the formation of the connections (Table 2). The most prominent indication was a higher level of significant propensities to make mutual connections. The connections had a tendency to be based on the homophily of contents of messages. However, the result of the EGRM questionnaire indicated no significant tendency for connections. The results indicate that gratitude is not a personal impression, but rather a form of interpersonal communication. Further analyses were conducted to clarify the relations among terms from different covariates.

### Comparison of terms

Results of the three analyses presented in Table 2 are arranged according to models, with dominant terms being selected for comparison.

Model 1 shows a null model with edges and reciprocated connections to process the continuous prediction. Model 2 includes terms with higher propensity in node-based covariate analysis. Model 3 includes terms with higher propensity in dyadic covariate analysis. Model 4 includes a term with higher propensity in structural covariate analysis.

### RQ2: What are online community characteristics? What structural connections of the network tend to emerge?

In Table 3, the term ‘mutual’ has the greatest propensity across all models. The homophily of the country and question number in models 3 and 4 also shows greater positive propensities. Apparently, the dyadic covariates governed crucially important effects on connections. Although a significant effect was found for students who posted many gratitude messages, the coefficient was small and was estimated as a limited contribution to connections. Whereas the baseline of a connection probability of model 4 was calculated as 0.00372, the log odds of the connection were greater when ‘mutual’, ‘nodecov.gratitude’, ‘nodematch.country’, and ‘nodematch.question’ were included. The probability was 0.444.

**Table 3:** Four models for positive affect relations in results of three covariate analyses

Term	Dependent variable: estimate			
Parameters	Model 1	Model 2	Model 3	Model 4
edges	− 4.558*** (0.089)	− 5.332*** (0.162)	− 5.600*** (0.181)	− 5.590*** (0.177)
mutual	3.515*** (0.276)	3.356***(0.273)	3.078*** (0.302)	3.101*** (0.299)
nodecov.access		0.004**(0.002)	0.004* (0.002)	0.003* (0.002)
nodecov.gratitude		0.176***(0.037)	0.208*** (0.047)	0.208*** (0.046)
nodematch.country			1.107*** (0.256)	1.110*** (0.257)
nodematch.question			0.910*** (0.145)	0.914*** (0.145)
absdiff.gratitude			− 0.063 (0.063)	− 0.065 (0.062)
gwesp.ITP				0.136 (0.192)

Upper, parameter estimate value; lower, parameter standard error

*p < 0.1; **p < 0.05; ***p < 0.01

The appendix shows an excerpt from messages within the dataset. It shows how students shared their answers to share related resources and their interpretations.

### Excerpt to exchange reciprocal messages with gratitude

### RQ3: How do messages of gratitude appear? To what extent are these messages deployed in the network?

Because the beginning of the communication was answering the question of a self-loop edge without gratitude, the homophily of gratitude intrinsically appeared in continuous contexts. In fact, 77.3% of the edges (133 of 172 connected edges) involved gratitude (blue edges in Fig. 3). Although the greatest and dominant propensity was observed in the formation of reciprocated messages, the students showed a greater tendency to express gratitude irrespective of whether their mindsets were framed as benefactors or beneficiaries. This relation was encapsulated as “the beneficent circle of gratitude,” where the increased gratitude of benefactor was also explained (White, 1999; Wood et al., 2010).

Howells proposed the following three challenges to establish gratitude in learning communication (Howells, 2014).

1. Systematic: conduct gratitude even when a sender has a low priority and time limitation to think
2. Conceptual: build resilience and show gratitude even when things do not go as planned
3. Reciprocity: practise gratitude even when there is no expectation of receiving anything in return

The student participants in this study were able to express their efforts to improve communication with gratitude behaviours. The following sentences from the appendix can be taken as an example of Howells’s systematic and reciprocity challenges.

*From S58 to S27: … It is clearly explained that the reason the ratio of government debt repayments in Venezuela’s foreign currency income is on the rise depends on oil exports*, *and that the ratio fluctuates because of fluctuations in oil prices*. *I understood it with gratitude because I had a chance to see your graph* ...

Although their knowledge sharing is evident in their communication, the expression of gratitude from S58 facilitated the further prosocial behaviour of the benefactor student S27. Increasing prosocial behaviour in the mutual connection by a beneficiary also reflects benefit to the benefactor (Grant & Dutton, 2012). The effect of prosocial behaviour in the online communication was explained by the notion of benevolence, which is unselfish and kind-hearted behaviour that earns the goodwill of other people (Solis, 2011; Yoshida, 2021). The response to the message above increased gratitude and communication and highlights both the conceptual and reciprocity challenges indicated by Howells (2014):

*From S27 to S58: … Mr*. *S58 taught me that I was able to realise that I had an ambiguous understanding*. *I’d like to express my gratitude to you …*

These excerpts demonstrate the network dynamics and the tendency of connections using effects of students’ behavioural dimensions.

### RQ4: How do messages with gratitude prompt reply messages?

Students made reciprocal messages of gratitude that included all three challenges. The reciprocal messages of gratitude were also explained in an earlier study of social media communication, in which the individual expected something in return for gratitude (Valociková & Velencei, 2020). However, in no case of our review of the dataset did a respondent request any reward from a questioner in our study. Academic communication is not always driven by reward. People experience a sense of meaning when they are viewing a story that highlights an act of moral excellence and virtue. Consequently, these altruistic experiences encourage people to participate in prosocial actions (Oliver et al., 2012). Although the feeling of gratitude is often a response to prosociality, its expression can also be a form of prosocial behaviour (Walker et al., 2016). Smooth communication includes altruism, which is crucially important for knowledge sharing (Chang & Chuang, 2011). Additionally, frequent presence results in reciprocity because feedback is presumed: users are more likely to share information in online environments (Valociková & Velencei, 2020). We were able to understand the characteristics of student communication and learning with gratitude.

The learning environment in the discussion forum for sharing messages was suitable to increase their experiences of gratitude and to develop prosocial behaviour in the students. In fact, all students had experiences of writing content; all students had experiences of gathering information and expressing individual opinions before communication. Therefore, their experiences reinforced active participation with gratitude in the ongoing discussion.

## Conclusion

As online education becomes more prevalent because of the spread of COVID-19, the effects of online communication on students are a matter of urgent concern. Although online message communication has been demonstrated to be a powerful environment for transforming behavioural change and for exchanging messages of gratitude, the findings reported herein indicated that the community was not structured by individual intimate relationships, but rather by academic connectedness based on the message contents. Similarly, the students’ post-questionnaire opinions did not influence their connections. Results of this study clarified some structural features of connections with messages of gratitude which were not described in earlier reports. Our results demonstrated that an online learning environment in which gratitude messages are disseminated cannot be analysed through personal impressions, but can be assessed through a form of interpersonal communication. A strong tendency for mutual connections was observed. These connections elicited messages of gratitude from both benefactors and beneficiaries. Our SNA was able to represent the emergence of prosocial behaviour in students. Consequently, our outcomes contribute to the communication and social media literature by showing how students’ connections, structural patterns, and mindsets associated with prosocial behaviour might shape their online sharing and information searching behaviours. We hope these results will encourage other researchers to investigate how increased gratitude can play a unique and important role in enhancing knowledge development in an online learning environment.

## Limitations and further research

The current research has some limitations. First, although we carefully selected a course topic that was deemed acceptable for evaluating scholarly communication, we did not attempt to generalise our findings to other academic courses. Additionally, we introduced an online database to students to collect data and to allow them to select countries for review in the online discussion. Different questions and countries might have been confusing for some students (e.g., identifying useful resources for each student), but active messaging was observed. This study demonstrated that the students were able to share the common goal of disparity and that they were able to implement it for their discussion. Nevertheless, no node attribute of student competencies related to ESD was introduced in this study. Future research should carefully examine the effects of the fundamentally important competencies of ESD. Second, we were unable to find any association between the results of the EGRM post-questionnaire and the results of ERGM. The difference in the investigation period and the dissonance between the impression and the behaviour are related. Therefore, future research should investigate why the questionnaire method failed to explain the observed behaviour of exchanging gratitude messages. Third, we detailed student performance in expressing gratitude depending on their formulated mindset as a benefactor or beneficiary. Although prosocial activities were evident in knowledge sharing by the students, future studies should also investigate which elements support their prosocial behaviour in lessons. We expect that social network analyses will be of great use in clarifying questions about gratitude such as those presented above.

## Data Availability

The datasets used and/or analysed during the current study are available from the author on reasonable request.

## Appendix

### Examples of messages in the online discussion forum

Note: The original messages were written in Japanese and were translated by the authors.

The names of students were replaced by assigned codes, as presented in Fig. 3

**Question 2 Venezuela**

**May 5, 2021 posted by S27**

I investigated Venezuela, which has a per-capita GDP of $16,054. [1] The graph below shows the secular change of Venezuela’s Gini coefficient (right axis) [2] and the ratio of government debt repayments within foreign currency income (left axis) [3] over the 10 years of 2005–2015.

The Gini coefficient shows a downward trend as a whole, although there are some fluctuations. However, the proportion of government debt repayments in foreign currency revenues has been increasing almost continuously. Comparing the shapes of the graphs, it can be seen that the overall trend is that as the Gini coefficient decreases, the proportion of government debt repayments in foreign currency income tends to increase.

Behind the fluctuations in these two indicators is the policy of the Chavez administration (1999–2013) [4]. President Chavez, who took office in the 1990s when poverty and inequality were worsening, emphasised social development and made large-scale investments in low-income earners. It is believed that this policy has reduced the disparity. However, the cost of such a policy was covered by abundant oil that could be extracted. It is thought that the ratio of government debt repayments in foreign currency revenues increased because the foreign currency obtained from oil exports was used to repay debts that increased with the expansion of policies. As described above, the two indicators are closely related to each other against the background of the administration's policy.

However, because the government relies on oil revenue, fluctuations in oil prices are expected to have a strong financial impact.

Looking at the actual data (quoted from [5]), the economic growth rate has shown a marked decline because of the decline in oil prices since around 2014.

Venezuela’s inflation rate [6] has shown a marked increase in recent years, reaching 255% in 2016.

The inequality that was once narrowing because of economic stagnation and rapid inflation is expected to widen considerably in the future. I think it is necessary for the international community to cooperate and urgently overcome the serious situation.

References

[1] https://data.worldbank.org/indicator/NY.GDP.PCAP.CD?locations=VE

[2] https://www.ide.go.jp/library/Japanese/Publish/Download/Report/2016/pdf/C16_ch02.pdf (p.31).

[3] https://data.worldbank.org/indicator/DT.TDS.DECT.EX.ZS?end=2015&locations=VE&start=2010

[4] http://hdl.handle.net/2344/00017049 (p.54–57).

[5] https://www.jstage.jst.go.jp/article/latinamericareport/35/1/35_35/_pdf/-char/ja (p.38).

[6] https://data.worldbank.org/indicator/FP.CPI.TOTL.ZG?locations=VE

**﻿Re Question 2 Venezuela**

**May 17, 2021 posted by S58**

I read your report.

It is clearly explained that the reason why the ratio of government debt repayments in Venezuela’s foreign currency income is on the rise depends on oil exports, and that the ratio fluctuates because of fluctuations in oil prices. I understood it with gratitude because I had a chance to see your graph. However, looking at the data, Venezuela’s oil exports are declining year by year, so I was not convinced that oil alone covers the country’s policies. Upon examination, I felt that Venezuela also earned foreign currency from these exports because of its abundance of natural gas and mineral resources.

What do you think, Mr. S27?

Reference

https://www.offshore-technology.com/comment/full-scale-oil-sanctions-venezuela-no-longer-necessary/?utm_source=Army%20Technology&utm_medium=website&utm_campaign=Must%20Read&utm_content=Image

**Re﻿ Question 2 Venezuela**

**May 17, 2021 posted by S27**

Thank you for your question.

As Mr. S58 said, it is impossible for oil alone to finance all policies. According to reference [4] p.57.

‘Oil income has been used to invest in infrastructure, social development, and the establishment of state-owned enterprises under the Chavez administration.’

It seems. From here, I think it is correct to interpret that oil has provided the additional funds needed for the development of the country. I’m sorry for the misleading expression.

Regarding the question of whether foreign currency is also obtained from other mineral resources, it is said that oil accounts for nearly 95% of the export breakdown in Venezuela [7]. Therefore, there is information that when crude oil prices fell and the government was forced to repay its external debt, there was a shortage of money for imports, causing a severe shortage of goods [7]. I think it’s a good example of how Venezuela depends on oil. Venezuela felt that there was an urgent need to find a way out of the oil industry.

Mr. S58 taught me that I was able to realise that I had an ambiguous understanding. I’d like to express my gratitude to you.

Reference

[7] https://media.monex.co.jp/articles/-/7933

## Abbreviations

ERGMSs: Exponential random graph models
GQ-6: The gratitude questionnaire 6
EGRM: Expression of gratitude in relationships measure
SNA: Social network analysis
ESD: Education for sustainable development
SDGs: Sustainable development goals
UNESCO: United Nations Educational, Scientific and Cultural Organization
GDP: Gross domestic product
PPP: Purchasing power parity
